# The impact of the COVID-19 pandemic on rates of adolescents receiving psychopharmacological medication in Austria

**DOI:** 10.1186/s13034-023-00684-x

**Published:** 2024-01-13

**Authors:** M. Otter, O. D. Kothgassner, L. Lepuschütz, S. Drahos, P. L. Plener

**Affiliations:** 1 Federation of Austrian Social Insurance Institutions, Kundmanngasse 21, 1030 Vienna, Austria; 2 Department of Child and Adolescent Psychiatry, Medical University Vienna, Waehringerguertel 18-20, 1090 Vienna, Austria; 3 Department of Child and Adolescent Psychiatry and Psychotherapy, University of Ulm, Steinhoevelstr. 5, 89075 Ulm, Germany

**Keywords:** COVID-19, Adolescents, Children, Psychopharmacology, Antidepressants, Antipsychotics, Benzodiazepines, SSRI

## Abstract

**Background:**

The COVID-19 pandemic has impacted many aspects of everyday life, including the (mental) healthcare system. An increase in depression and anxiety symptoms has been reported worldwide, and is particularly pronounced in females and young people. We aimed to evaluate changes in prescription rates for psychopharmacological medication, which is often used to treat depression and anxiety.

**Method:**

Based on data from the Austrian public health insurance institutions, we conducted an interrupted time series analysis of antidepressants and antipsychotics, comparing prescription rate developments before and throughout the COVID-19 pandemic (2013 to 2021), with a special focus on adolescents (10–19 years) in comparison to the general population. Data were based on all public prescriptions in the outpatient sector nationwide. Age- and sex-stratified time-series models were fitted to the pre-COVID period (first quarter (Q1) of 2013 to second quarter (Q2) of 2020). These were used to generate forecasts for the period from the third quarter (Q3) of 2020 to the fourth quarter (Q4) of 2021, which were subsequently compared to observed developments in order to assess significant deviations from the forecasted development paths.

**Results:**

For the majority of the evaluated period, we found a significant excess of antidepressant prescriptions among both male and female adolescents (10–14 and 15–19 years) compared to the forecasted development path, while the general population was mostly within 97.5% confidence intervals of the forecasts. Regarding antipsychotics, the interrupted time series analysis revealed a significant excess in the group of female adolescents in almost all quarters, which was especially pronounced in the 15–19 age group. Prescription rates of antipsychotics in the general population only showed a significant excess in two quarters.

**Conclusion:**

Increased rates of adolescents receiving psychopharmacological treatment echo the epidemiological trends of an increase in depression and anxiety symptoms reported in the literature. This increase is especially pronounced in female adolescents.

## Background

The COVID-19 pandemic has exerted a profound impact on society, affecting employment, schooling, healthcare and many other aspects of everyday life. In terms of mental health, increasing rates of symptoms of depression, anxiety, sleep problems, and other conditions have been reported across different continents, demonstrating the global consequences of the pandemic [7] According to an estimate based on various data sources worldwide, between January 2020 and January 2021, cases of major depressive disorder increased by 27.6% and cases of anxiety disorders by 25.6%, and this increase was particularly pronounced among women [7]. With respect to different age groups, results of studies conducted early on in the pandemic as well as studies on previous epidemics such as severe acute respiratory syndrome (SARS) or Ebola suggested that adolescents and young adults aged between 15 and 25 years showed the sharpest increase in mental health problems [4, 15]. This finding has been replicated over the further course of the pandemic, with adolescents in the transitional stage between childhood and adulthood showing elevated rates of symptoms of depression and anxiety compared to other age groups [7]. A recent meta-analysis encompassing 29 studies including children and adolescents reported pooled prevalence estimates of 25.2% for depressive symptoms and 20.5% for anxiety symptoms [33].

A higher burden of the pandemic on mental health in young people has likewise been reported in Austria. For instance, an online survey conducted between February and March 2021 found higher rates of symptoms of depression and anxiety than in the adult population among adolescent samples in school [28] and in apprenticeships [11], with 55% of adolescents showing moderate depressive symptoms and 47% scoring above the cut-off for anxiety symptoms [28]. Moreover, in a further survey conducted between September and November 2021 using the same methodology, the rate of Austrian adolescents reporting mental health problems remained high, with 58% reporting depressive symptoms and 46% reporting anxiety symptoms [10].

The increasing rates of mental health problems have also led to more contacts with the mental healthcare sector. A Danish cohort study of young people (age 5–24 years) reported an overall relative increase of incident psychiatric diagnoses of 5% during the COVID-19 pandemic compared to the expected rates [3]. While suicide rates remained stable during the first and subsequent months of the pandemic until June 2021 [29, 30], emergency department visits due to suicidal ideation and suicide attempts increased among adolescents [5, 21, 23, 43]. Only a small number of studies have explored the impact of the COVID-19 pandemic on rates of prescription of psychotropic drugs. A recent study of the general population conducted using Austrian social insurance data focused on prescriptions in 2020, and found no significant increase in psychopharmacological prescriptions during the first lockdowns in 2020 [40]. By contrast, focusing on a younger age population, a Danish nationwide cohort study of 5–24 year-old patients reported an increase in incident use of psychopharmacological interventions, which was especially pronounced in the age group between 12 and 17 years [3].

As the COVID-19 pandemic has been associated with an increase in mental health problems throughout society, with a particularly steep increase in adolescents, we sought to assess changes in psychopharmacological medication prescription rates in different age groups before and after the pandemic-related restriction measures. In view of the literature reporting increased rates of symptoms of depression, anxiety and sleep disorders in adolescents, we undertook a detailed analysis of the group of 10–19 year-olds.

The specific focus was on medication classes that are likely to be used in the treatment of children and adolescents with depression and anxiety, namely antidepressants and antipsychotics. We further aimed to track the prescription rates for these classes of psychotropic drugs in Austrian minors throughout the pandemic and compare them to expected rates based on former, pre-pandemic prescription patterns.

## Methods

### Data

The analysis was performed based on routine data from the umbrella organization of Austrian social insurance institutions (the Federation of Austrian Social Insurance Institutions), which records data for accounting purposes. The dataset comprises data on all people insured under the statutory social insurance, i.e. 98.5% of the Austrian population, corresponding to approximately 8.82 million people [8]. It includes all public prescriptions in the outpatient sector that were collected in pharmacies or from dispensing doctors nationwide within the given time frame, the first quarter of 2013 (Q1 2013) to the fourth quarter of 2021 (Q4 2021). Medication obtained through private outpatient prescriptions or over-the-counter medication are not included, and data from the hospital sector are also not included as the outpatient and inpatient sectors are run by different entities in the Austrian healthcare system. We used quarterly prescription rates of the following medications and respective Anatomical Therapeutic Chemical Codes (ATC Codes): antidepressants (ATC Code N06A) and antipsychotics (ATC Code N05A) for the analysis. A further examination, which is beyond the scope of the present article, revealed that non-selective monoamine reuptake inhibitors (N06AA), monoamine oxidase A inhibitors (N06AG), and the category other antidepressants (N06AX) had very small prescription rates among adolescents, especially among the younger group of 10–14 year-olds, with below 100 prescriptions per quarter on average in most groups and two medication groups showing 0 prescriptions in all adolescent groups.

The prescription rate was defined, based on [32], as the proportion of insurees who had at least one prescription dispensed in a given quarter within a specific ATC group per 1000 insurees in the same age and sex group (therefore representing an age- and sex-adjusted rate). An insuree receiving the respective medication is counted distinctly per quarter, meaning he or she is only counted once per quarter even if the prescribed medication is received several times.

### Statistical analysis

An interrupted time series analysis (ITS) was performed to test for the influence of the pandemic on prescription rates for the relevant medication groups. Measures to restrict the spread of COVID-19, such as lockdowns, clearly divided the observation period into a pre- and post-period, with an intervention point dividing these periods chosen in the second quarter of 2020. For the population group of interest, Austrian adolescents, the longest period of home schooling/distance learning occurred from the beginning of November 2020 to 8 February 2021, with the first (shorter) lockdown period taking place in March 2020. Since the first lockdown was restricted to a few weeks, we hypothesized an increase in the prescription rate of antidepressants, antipsychotics, and benzodiazepines starting in Q3 of 2020. To account for underlying short- and long-term trends in the data, pre-existing trends in prescription rates must be taken into account, such that a steady or a seasonal increase in rates is not attributed to the pandemic. ITS controls for issues such as trends and seasonality by longitudinally tracking outcomes before and after an intervention [35].

**Fig. 1:**
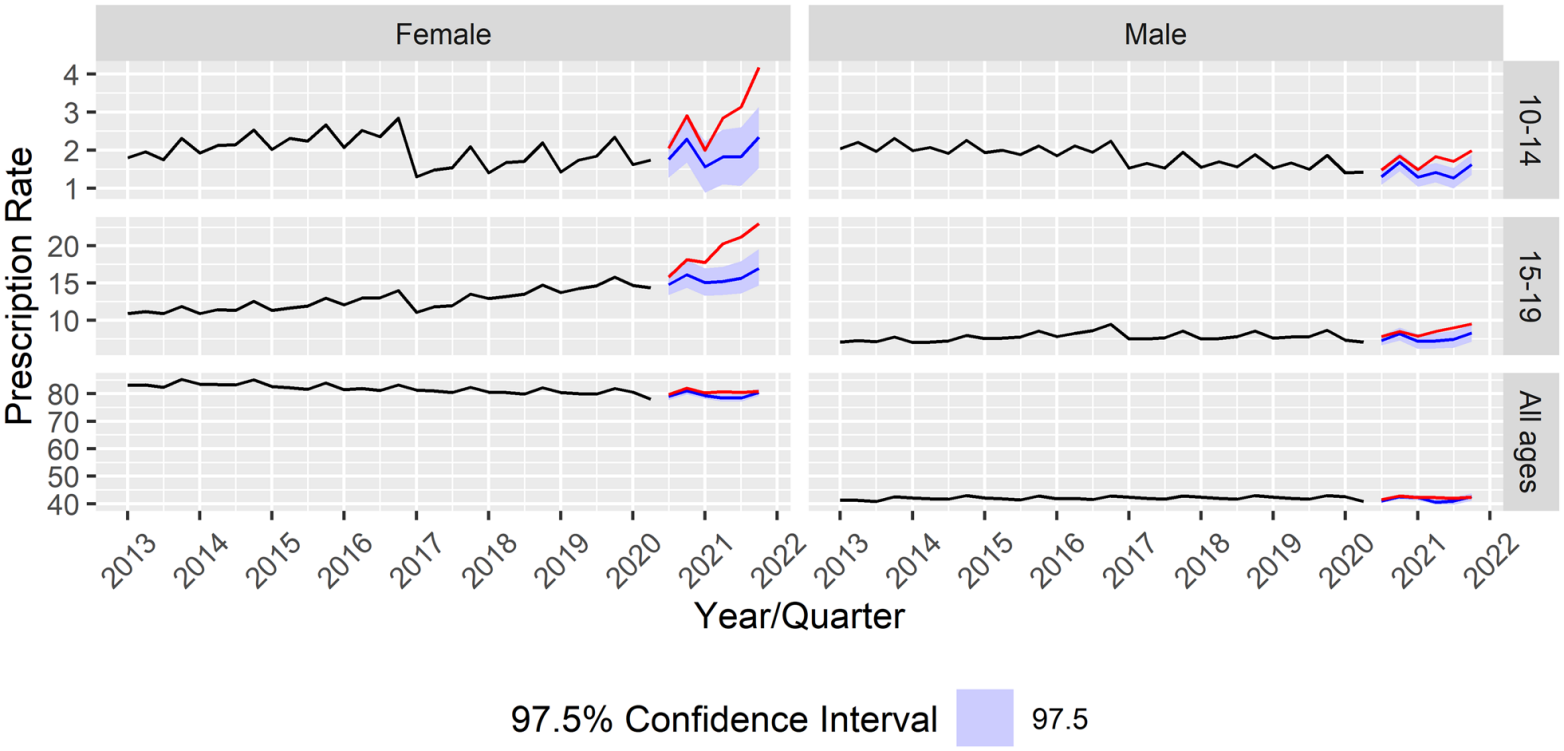
Prescription rate developments and forecasts for antidepressants stratified by age and sex group. Age groups (10–14 years, 15–19 years, all age groups combined, including adults) according to gender from Quarter 1 (Q1) of 2013 to Quarter 4 (Q4) of 2021

**Fig. 2:**
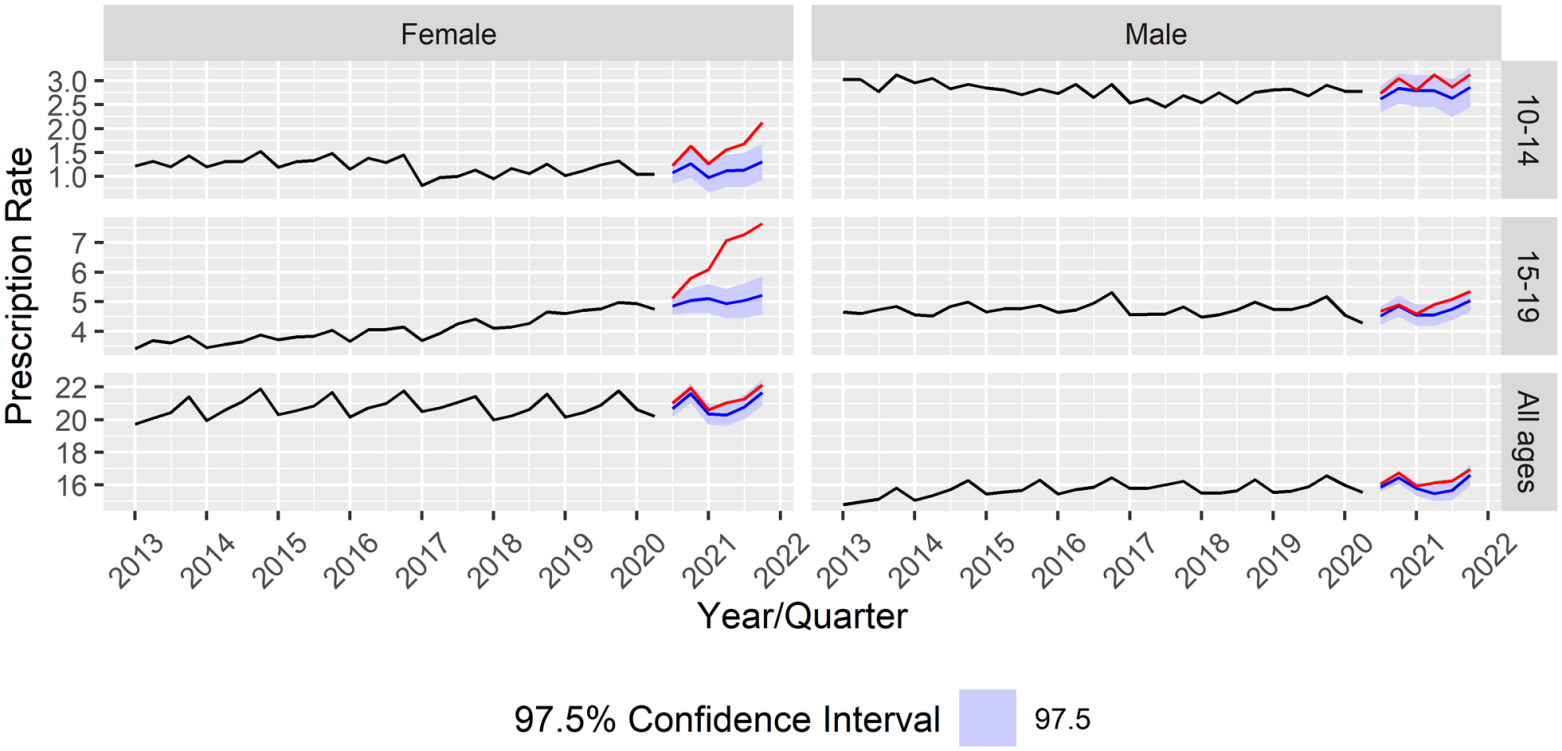
Prescription rate developments and forecasts for antipsychotics stratified by age and sex group. Age groups (10–14 years, 15–19 years, all age groups combined, including adults) according to gender from Quarter 1 (Q1) of 2013 to Quarter 4 (Q4) of 2021

The dataset for each medication was split into three age groups (10–14 years, 15–19 years, all ages combined, comprising every person insured in Austria, regardless of age) and two genders (male, female), resulting in 18 individual age- and sex-stratified time series. The time series data for each group was modelled from the start of the dataset in early 2013 to the pandemic-related restrictions in the second quarter of 2020 (Q2 2020). This was done to forecast confidence intervals at the 97.5% level based on these models for the post-restriction period from the third quarter of 2020 (Q3 2020) until the end of 2021. These confidence intervals represent expected development paths, based on time series developments only prior to the pandemic-related restrictions, and provide the best projection of what would have happened in the absence of the restrictions. The forecasts were subsequently compared to post-restriction observations in order to analyze deviations (see Figs. 1, 2). The forecasts were obtained using seasonal autoregressive integrated moving average (ARIMA) models. The analysis was performed using R [31], the packages tidyverse, lubridate and tsibble for transformation of the dataset [18, 38, 39], and the package fable to fit and select ARIMA models [27]. Each model was fit by choosing the optimal model according to the Fable package automatic model selection (with transformations applied according to prespecification), which uses a variation of the Hyndman-Khandakar algorithm selecting for the smallest AICc value [19, 20].

As the dataset provided by the Federation of Austrian Social Insurance Institutions consisted of accumulated data without the possibility of identifying individualized data, a waiver was received from the ethical review committee of the Medical University of Vienna.

## Results

The results revealed an increase in the prescription rate of antidepressants and antipsychotics, which was especially pronounced in the age groups 10–14 and 15–19 years, and a steeper increase among female adolescents (see Table 1).

**Table 1:** Absolute and relative change in the prescription rate between Quarter 3 (Q3) of 2020 and Quarter 4 (Q4) of 2021

Medication	Age	Sex	2020 3rd Quarter	2021 4th quarter	Change in Percent %
Antidepressants					
Antidepressants	10–14	Female	2.05	4.18	103.54
Antidepressants	10–14	Male	1.48	1.99	34.69
Antidepressants	15–19	Female	15.83	23.03	45.47
Antidepressants	15–19	Male	7.81	9.53	22.04
Antidepressants	All ages	Female	79.77	80.96	1.49
Antidepressants	All ages	Male	41.52	42.37	2.04
Antipsychotics					
Antipsychotics	10–14	Female	1.22	2.13	74.26
Antipsychotics	10–14	Male	2.73	3.14	14.78
Antipsychotics	15–19	Female	5.12	7.65	49.32
Antipsychotics	15–19	Male	4.68	5.36	14.40
Antipsychotics	All ages	Female	21.01	22.14	5.38
Antipsychotics	All ages	Male	16.04	16.94	5.61

This table displays the change in the respective prescription rates between the third quarter of 2020, the beginning of the effect, and the fourth quarter of 2021, the end of the observation period

### Antidepressants

Within the group of antidepressants, prescription rates among males in the 10–14- and 15–19-year age groups exceeded the 97.5% confidence intervals in three out of six observed quarters, while prescription rates for females in those age groups significantly exceeded predictions in five (10–14 year-olds) and six (15–19 year-olds) out of six observed quarters, respectively (see Fig. 1). This difference was even more pronounced when considering the differences in relative changes. The growth in prescription rates from Q3 2020 to Q4 2021 was 103.5% for 10–14-year-old females and 45.5% for 15–19-year-old females, while the growth in prescription rates for their male counterparts lay at only 34.7% and 22%, respectively. When combining all age groups, prescription rates only differed significantly from the model forecasts in one quarter for male patients and two quarters for female patients. Thus, increases more often exceeded model forecasts in female than in male patient groups, and these excesses were much more common in younger age groups than in the general population.

### Antipsychotics

A similar pattern emerged regarding the development of antipsychotic prescription rates during the observation period Q3 2020–Q4 2021. Here, the gender gap among the younger age groups was even more pronounced than for antidepressants. As can be seen in Fig. 2, forecasts were exceeded in four out of six quarters in 10–14 year-old females and in five out of six quarters in 15–19-year-old females, while male adolescents of both age groups showed no excesses throughout the observation period. This difference is also visible in the relative growth rates between Q3 2020 and Q4 2021, with prescription rates growing by 74.3% in 10–14 year-old females and by 49.3% in 15–19 year-old females, while their male counterparts showed much smaller growth rates (14.8% and 14.4%, respectively).

## Discussion

Our analysis focused on the influence of the COVID-19 pandemic on prescription patterns of common psychotropic drugs with a specific focus on adolescents. Given the reported high burden of mental health problems among adolescents during the pandemic [33, 42], we specifically examined the age groups of 10–14-year-olds and 15–19-year-olds regarding prescription rates of antidepressants and antipsychotics.

### Antidepressants

Antidepressant prescription rates showed a steep upward trend among females aged 10–19 years, which were significantly above model predictions in four (10–14 year-olds) and five (15–19 year-olds) out of six quarters, respectively. No comparable trend was found in the general population (all age groups combined), with only individual quarters significantly exceeding model predictions. Pre-pandemic rates showed a high degree of stability when looking at the pattern from 2013 onwards. A sharp decline was observed between 2016 and 2017. This coincided with a debate about the efficacy of SSRIs in adolescents based on a meta-analysis [6], which received broad media coverage in Austria and Germany [22, 36]. Shortly after the start of the pandemic, prescription rates for antidepressants began to increase steadily. Given that SSRIs are recommended as part of the treatment strategy for depression and anxiety in national guidelines [1, 3]; (AWMF, 2013), these elevated prescription rates can be interpreted as part of an increased treatment effort to counter the rising mental health problems throughout the COVID-19 pandemic.

The increase in prescription rates was more pronounced in female than in male patients, with the steepest increase in antidepressant prescription rates found in female adolescents. This is consistent with findings of increasing rates of depressive symptoms reported worldwide. For instance, in a meta-analysis of 29 studies encompassing data from 80,879 children and adolescents globally, Racine et al., [33] found a pooled prevalence rate of 25.2% for clinically elevated symptoms of depression, with higher rates of depressive symptoms reported in females. Recently, another meta-analysis based on 53 longitudinal studies from 12 countries also reported an increase in depressive symptoms in children and adolescents, which was stronger in female than in male participants [24]. Besides data from international samples, studies on the mental health of Austrian adolescents during the pandemic point in the same direction, with an increase in depression and anxiety symptoms that was especially pronounced in female participants [10, 28].

### Antipsychotics

From all evaluated age groups, the strongest increase in prescription rates was observed in 15–19-year-old females. As the use of antipsychotics in minors is only licensed in Austria for the treatment of bipolar disorder, schizophrenia, or major impulsive aggressive behavior, these rising rates, particularly among females, are highly interesting. Despite research demonstrating a particularly severe impact of the COVID-19 pandemic on people with schizophrenia [16], few studies have explored a potential increase in first episodes of schizophrenia. One study, conducted in Australia, reported an increase in first-episode admissions for schizophrenia among young people following the introduction of lockdown measures [26]. Additionally, while the pandemic has been linked to first presentations of manic episodes [34], evidence regarding potential increases in bipolar disorders is lacking. Atypical antipsychotics have been mentioned as part of an augmentation regime in the treatment of resistant depression [14, 37], but are only suggested for the treatment of psychotic depression in minors [25]. Therefore, the rise in prescriptions of antipsychotics might also be interpreted as indicating an increased off-label use for the treatment of depression in minors. Furthermore, some antipsychotics are used as an off-label medication for severe anorexia nervosa [17]. As an increase in eating disorders has been reported throughout the pandemic [13], increasing prescription rates of antipsychotic prescriptions may echo an increasing clinical demand in this field. This might further explain the higher prescription rates among females found in the present study, as the rise in eating disorders is especially pronounced among female adolescents [13], potentially accompanied by increased psychopharmacological treatment. Unfortunately, we are unable to link prescriptions to diagnoses, as diagnostic ICD-10 codes are not currently available from the outpatient sector, from which this dataset is derived. Therefore, it can only be hypothesized that the use of antipsychotics in female adolescents corresponds to increased rates of treatment for depression, eating disorders, or other symptoms or disorders such as sleep problems.

Overall, we were able to demonstrate an increase in prescriptions of antidepressants and antipsychotics in adolescents, which was especially pronounced in females. The observed trends are in line with increasing levels of symptoms of depression and anxiety during the COVID-19 pandemic as reported globally [23, 33] and in Austria [10, 28].

Data regarding prescription rates of psychopharmacological agents in children and adolescents during the COVID-19 pandemic are scarce, although a report issued by the German health insurance company DAK described a substantial increase in antidepressant prescriptions in adolescent females between 2019 and 2021 (+ 30% in 10–14-year-olds and + 65% in 15–17-year-olds in those diagnosed with a depressive disorder) [41]

Our findings are consistent with this reported increase in antidepressant prescriptions, but extend further than these datasets in terms of the population covered. For example, while the DAK report includes data from 5.7% of German children and adolescents [41], our dataset includes approximately 858,000 Austrian adolescents, representing about 99.4% of the Austrian population in this age group. Furthermore, we were able to analyze two different medication groups (antidepressants and antipsychotics).

A study from the US using data from the IQVIA health insurance company (encompassing roughly 8.9 million minors aged between 2 and 17 years) analyzed monthly prescription rates of ADHD medication, antidepressants, antipsychotics, and mood stabilizers between January 2019 and September 2020. The results revealed a spike in overall psychopharmacological prescriptions in April 2020, which subsequently returned to normal, pre-pandemic levels [2]. This trend is in line with the patterns assessed in our data, although it appears that the upward trend in the US sample is more short-lived than in our study. This might be explained by differences in the availability of mental health practitioners, different COVID-19 restrictions, and also different time frames of the respective analyses. A cohort study of Danish youth also reported an increase in incident prescriptions of psychotropic medication between March 2020 and June 2022, which was most prominent in the 12–24 years age group [3]. Interestingly, the rise in prescriptions was seen for all groups of psychotropic drugs, including hypnotics and sedatives, psychostimulants, antidepressants, and antipsychotics, but not in the group of anxiolytics. A recent study from Austria [40], analyzing a shorter time frame of 2020 only, observed no significant changes in defined daily doses of psychopharmacological drugs between 2019 and 2020 in any age group. The differences from our findings are likely due to the different time intervals examined in the two studies: While the present study observed cumulative effects over six quarters, the study by [40] focused on medication prescriptions during the national lockdowns in 2020. Nevertheless, it should be noted that in the study by [40], the age group of 10–20-year-olds likewise showed the largest percentage increase in psychopharmacological prescriptions of all age groups.

## Limitations

Despite several strengths of the present study, such as a dataset of 8.82 million insured persons in Austria, representing about 98.5% of the Austrian population, several limitations need to be addressed. First, the use of anonymous, aggregated data limited the depth of analysis. However, given the size of the dataset, we feel that this study contributes interesting information for healthcare planning. Second, not all of these 8.82 million people are necessarily living and registered in Austria and therefore part of the Austrian census, while some people who are registered in Austria and therefore part of the Austrian population are insured in neighboring countries. This is mostly the case with cross-border commuters. It is difficult to quantify the exact number of people who are insured but not part of the Austrian census, although the impact on the sample should be minimal given the large scale of the dataset; at most, it should account for two percentage points of the whole dataset and likely even lower for the age groups of interest, namely adolescents. Third, data on medicines below the Austrian prescription fee threshold (€6.50 in 2021 and adjusted annually) are not collected in the central dataset, as patients pay for prescriptions below this threshold themselves (with the exception of individuals or households that are exempted from prescription fees due to low income or high total healthcare costs) [9]. The medications in our dataset are affected by this effect to different degrees, with antipsychotics and antidepressants being mostly above the threshold (85% and 59%, respectively).

With regard to our statistical approach, two points warrant further attention. First, it is difficult to determine the precise timing of the pandemic-related restrictions, as there were several lockdowns on the national level as well as additional federal measures and restrictions, which varied strongly over time. Additionally, there were other restrictions and lockdowns on a regional level which only affected parts of the Austrian population. Second, we expected the effect to occur with a time lag, given that a certain delay can be expected with regard to a possible impact on mental health and subsequent help-seeking followed by prescriptions (as symptoms take time to develop and healthcare professionals need to be sought for treatment). To test the robustness of our results regarding the timing of the pandemic-related restrictions, we used the same modelling procedure but chose two alternative, but in our opinion less fitting, restriction time specifications: the first quarter of 2020 and the third quarter of 2020. In these different specifications, the overall patterns stayed the same. While some quarters lost significance and others gained significance, this did not affect the overall structure of the results.

## Conclusion

Data from the Federation of Austrian Social Insurance Institutions show an increase in prescriptions of antidepressants and antipsychotics throughout the pandemic, which was especially pronounced in female adolescents. The increasing rates of depression and anxiety symptoms that have been reported globally and in Austria appear to be associated with increased use of corresponding psychotropic medication. However, the increasing rates of prescriptions of antipsychotics warrant further attention and analysis, given that the evidence for their use in this age group is limited.

## Data Availability

The data is owned by the Federation of Austrian Social Insurance Institutions. Requests for data can be sent to the first author.
